# Advanced Ultrasound Techniques for Neuroimaging in Pediatric Critical Care: A Review

**DOI:** 10.3390/children9020170

**Published:** 2022-01-30

**Authors:** Colbey W. Freeman, Misun Hwang

**Affiliations:** 1 Department of Radiology, University of Pennsylvania Health System, Philadelphia, PA 19104, USA; Colbey.Freeman@pennmedicine.upenn.edu; 2 Department of Radiology, Children’s Hospital of Philadelphia, Philadelphia, PA 19104, USA

**Keywords:** contrast-enhanced ultrasound, microvascular imaging, elastography

## Abstract

Because of its portability, safety profile, and accessibility, ultrasound has been integral in pediatric neuroimaging. While conventional B-mode and Doppler ultrasound provide anatomic and limited flow information, new and developing advanced ultrasound techniques are facilitating real-time visualization of brain perfusion, microvascular flow, and changes in tissue stiffness in the brain. These techniques, which include contrast-enhanced ultrasound, microvascular imaging, and elastography, are providing new insights into and new methods of evaluating pathologies affecting children requiring critical care, including hypoxic–ischemic encephalopathy, stroke, and hydrocephalus. This review introduces advanced neurosonography techniques and their clinical applications in pediatric neurocritical care.

## 1. Introduction

Because of its portability, safety profile, and accessibility, ultrasound has been integral in pediatric neuroimaging, particularly in the critical care setting. Conventional B-mode or grayscale imaging provides helpful anatomic information, but does not offer insight into the pathophysiologic processes underlying changes in anatomy and morphology. Doppler ultrasound, both color and power, visualizes and measures relatively high-rate flow, usually of blood. While this provides functional information grayscale imaging cannot, conventional Doppler ultrasound is insensitive to slow flow because of limitations from its method of removing background tissue clutter.

New and developing advanced ultrasound techniques provide physiologic information that was previously unavailable at the bedside and required cross-sectional imaging modalities with higher cost and logistical challenges, such as transport of critically ill patients and sedation. Contrast-enhanced ultrasound (CEUS), microvascular imaging (MVI), and elastography facilitate real-time visualization of brain perfusion, microvascular flow, and changes in tissue stiffness in the brain. These techniques are providing new insights into and potential new methods of evaluating pathologies affecting children in critical care, including hypoxic–ischemic encephalopathy (HIE), stroke, and hydrocephalus. While they have so far been primarily employed in research settings, they have the potential to affect clinical care for critically ill patients.

In this narrative review, we describe the technical foundations of these advanced ultrasound techniques and their applicability to pediatric neuroimaging. We also illustrate potential imaging findings in relevant pathologies. Ultimately, we hope to inform readers of recent innovations in neurosonographic evaluation of children requiring critical care.

## 2. Contrast-Enhanced Ultrasound

Contrast-enhanced ultrasound is an advanced ultrasound technique that utilizes microbubbles comprising a shell, often composed of lipid, encapsulating a gaseous core. The microbubbles measure approximately 1–10 µm in diameter, typically closer to 2–3 µm, which is smaller than red blood cells, which measure approximately 7–8 µm. While other techniques, such as conventional color and power doppler, permit the evaluation of macrovascular flow, the small size of microbubbles allows them to enter capillaries and other small vessels, visualizing the microvasculature. The gas within the microbubbles creates an acoustically reflective surface that generates echoes detectable by ultrasound. Contrast is typically introduced via venous access and can be injected as a bolus or as a slower continuous infusion. The microbubbles remain in the brain for up to 10 min and are predominantly eliminated through the lungs. In infants, open fontanelles provide natural acoustic windows. In older children, other windows, such as the transtemporal window, may be used.

When used intravascularly, ultrasound contrast is administered intravenously, not intra-arterially. Contrast can be administered as either a bolus injection or an infusion via an infusion pump or by gravity. The latter is often performed using a flash-replenishment method wherein a high mechanical index pulse destroys existing microbubbles and contrast is allowed to reenter the tissue of interest. There is no standard dosing regimen for either approach. However, bolus doses up to 6.25 mL and infusion rates of 0.80 mL/second have been used for visualization of brain perfusion [1,2]. For the evaluation of hypoxic–ischemic injury, a bolus dose of 0.03 mL/kg has previously been described [3]. Bolus doses can be repeated once for evaluation of additional findings or for confirming findings on the initial exam [2]. Boluses should be followed by a flush of normal saline. Cine clips are typically performed for 45 s to 1 min for either the bolus or infusion methods. Delayed static and cine clips may be obtained as clinically indicated, as certain pathologies such as hypoxic–ischemic injury can exhibit delayed washout of contrast [4,5]. Longer clips are performed if particle tracking techniques are used wherein microbubbles are tracked across thousands of frame rates to resolve detailed morphologic and functional microvascular information. Contrast injection is typically performed via a peripheral intravenous line but may also occur through a central venous line. Smaller needles or catheters may result in microbubble destruction and reduce enhancement, but the gauge at which this occurs depends on the specific contrast agent used [6].

CEUS requires a low mechanical index (<0.2) contrast mode that is available on many commercial ultrasound scanners. The mechanical index of typical B-mode or color Doppler (>1) is too high and leads to microbubble destruction. A curved transducer between 2 and 8 MHz [2] is preferred, as this frequency range coincides with the resonant frequency of microbubbles. A sector transducer can also be used, and linear transducers can be used to image more superficial structures. Note, however, that a slightly increased dosing and/or mechanical index may be necessary for CEUS applications using linear transducers [7,8].

Ultrasound contrast is widely regarded as safe for pediatric usage. The contrast agents themselves cause few reactions in children [9]. In a study covering 5079 intravesicle and intravenous injections of ultrasound contrast in children ranging from neonates to 18-year-olds, reported reactions occurred in only 0.52% of studies and included skin reactions, taste alterations, and hyperventilation [10]. Other side-effects, such as lightheadedness, headache, and nausea, have been reported in adults. Allergic-like or hypersensitivity reactions are a potential risk of ultrasound contrast administration and are exceedingly rare. In adults, a large-scale study following over 30,000 patients identified only two cases of anaphylaxis [11]. A recently published review of safety of ultrasound contrast agents in children calculated that the overall risk of any reaction is 1.20% and the risk of severe reaction 0.22%, lower than the 0.6% of computed tomography (CT) iodinated contrast [12]. Ultrasound contrast agents should be avoided in patients with known sensitivities to the composing ingredients, specifically polyethylene glycol. The mechanical index for CEUS is set far below the maximum safe levels dictated by the Federal Drug Administration of the United States and other organizations by default on most ultrasound machines. However, a destructive pulse or flash uses a higher mechanical index, 1.0 or higher, and safety data for use in humans are limited.

**Figure 1:**
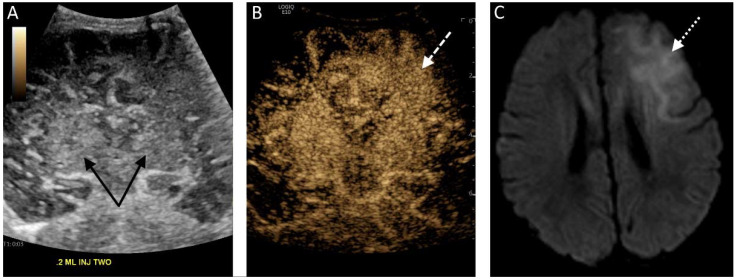
(**A**) Midcoronal grayscale ultrasound image of a 2 day old newborn’s brain through the deep gray nuclei (black solid arrows). (**B**) The contrast-enhanced image acquired 5 s after a second bolus injection in the same plane demonstrates enhancement of the bilateral deep gray nuclei but also asymmetric hyperenhancement of the left frontal lobe (white dashed arrow), representing luxury perfusion in an area of infarct. (**C**) A diffusion-weighted MRI sequence confirms the presence of diffusion restriction in the left frontal lobe (white dotted arrow) consistent with left frontal infarct.

In multiple countries, ultrasound contrast agents have been approved for use in echocardiography, liver lesion visualization and characterization, and evaluation of vesicoureteral reflux. However, ultrasound contrast has additional off-label applications for which it is not currently approved but may be useful in the pediatric critical care setting, such as visualization of brain perfusion (Figure 1). Brain perfusion evaluation would otherwise require the use of cross-sectional imaging, such as arterial spin labeling magnetic resonance imaging (MRI), which is costly, has more limited availability, and is logistically challenging for children requiring critical care. CEUS can be performed at the bedside, is more widely available, and does not require sedation.

**Figure 2:**
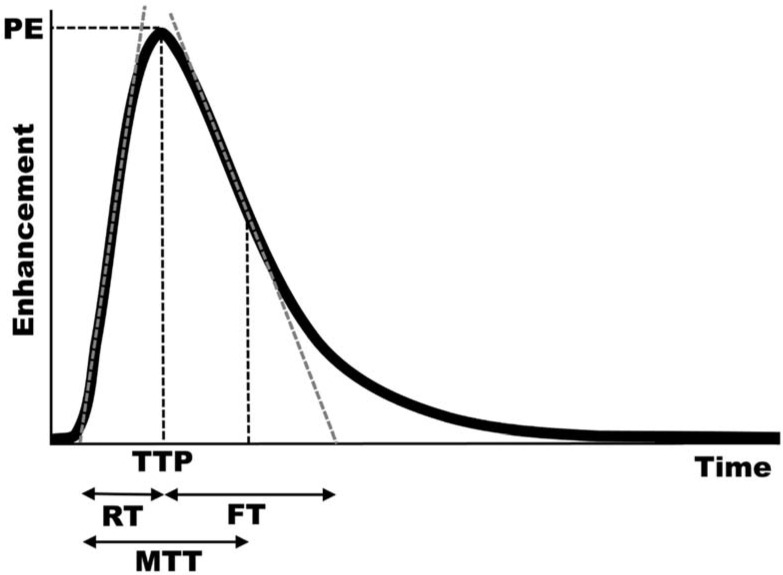
Typical enhancement curve of brain perfusion after bolus administration. Peak enhancement (PE) is the maximum enhancement in the region of interest, and the time to that peak enhancement (TTP) represents an additional measure of perfusion. The maximum slope in the wash-in phase (gray dashed line on the wash-in curve) and maximum slope of the wash-out phase (gray dashed line on the wash-out curve) can be calculated, and the intersections of those lines at the *x*-axis can be used to calculate rise (RT) and fall (FT) times. Mean transit time (MTT) is calculated as the time between 50% peak enhancement on the wash-out curve and the intersection of the wash-in slope with the *x*-axis.

CEUS can provide both quantitative and qualitative evaluation of brain perfusion. There are many quantitative measures of brain perfusion on time–intensity curves from CEUS using both bolus (Figure 2) and infusion techniques, including peak enhancement, time to peak, rise and fall times, wash-in and wash-out times, and area under the curve. With the bolus technique, peak enhancement is the maximum enhancement in the region of interest, usually expressed in arbitrary units, and the time to that peak enhancement represents an additional measure of perfusion. The maximum slopes of the wash-in and wash-out phases can be calculated, and the intersections of those lines at the *x*-axis can be used to calculate rise and fall times. Mean transit time is calculated as the time between 50% peak enhancement on the wash-out curve and the intersection of the wash-in slope with the *x*-axis. Area under the curve represents the entirety of the area under the time–intensity curve from wash-in through wash-out.

A more advanced quantitative method in CEUS utilizes particle image velocimetry, or particle or microbubble tracking. Using this technique, the vectors and paths of individual or small groups of microbubbles can be ascertained. Although highly experimental, this technique may be able to detect more subtle changes in perfusion than the typical parameters employed in CEUS and can be used to map microvasculature. Preliminary data demonstrate that cerebral microvasculature can serve as an imaging marker for intracranial pressure and brain ischemia in hydrocephalus [13]. Some requirements for microbubble tracking limit its use to experimental settings at this time. First, current microbubble tracking techniques require a relatively high frame rate, often above 30 frames per second and sometimes as high as 45 or 60 frames per second depending on the sensitivity desired. The field of view on modern ultrasound machines that provides this framerate may be small, limiting imaging to a specific part of the brain. With further advancements in computational speed and machine learning integration, real-time feedback and the use of such advanced post-processing methods at the bedside may be feasible in the future.

Moreover, the use of these measures is currently limited by the lack of normative data, and large-scale studies are needed to address this gap in the literature. Nevertheless, conclusions can still be drawn based on the acquisition of multiple studies at different timepoints, and, like many studies in radiology, identification of gross global or focal abnormalities in perfusion is possible with experience with the modality. It is important for the individual performing the exam to have sufficient experience, as CEUS, like other ultrasounds, is dependent on the technical skill of the operator. Using the bolus technique, enhancement may be affected by injection speed, hemodynamic parameters, external support (i.e., extracorporeal membrane oxygenation), and/or medications such as pressors. As previously noted, IV gauge may also affect the degree of enhancement. Therefore, standardization of technique is important to ensure reproducibility and permit comparison between exams. The visualization of brain perfusion is useful in multiple pathologies affecting pediatric patients requiring critical care, including HIE and stroke [2,14]. A study with piglets showed a correlation between perfusion on CEUS and dynamic susceptibility contrast MRI in the setting of perinatal hypoxia [15]. In an early small-scale study with 11 infants with ischemic and other lesions on MRI, CEUS had a sensitivity of 88.9% for the MRI findings [16]. In subsequent years, additional studies have shown the utility of CEUS for the evaluation of HIE both with qualitative detection of focal, multifocal, or diffuse perfusion abnormalities and with changes in quantitative measures [3,4,14,17].

**Figure 3:**
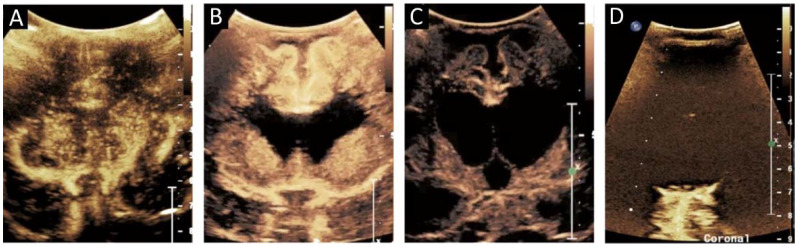
(**A**) Midcoronal contrast-enhanced ultrasound image of a 1 month old infant brain obtained 15 s after contrast administration reveals greater perfusion in the deep gray nuclei than in the more peripherally located white matter and the most peripheral cortex, normal for age. (**B**) A midcoronal brain image of a 10-month-old after cardiac arrest status post extracorporeal membrane oxygenation reveals increased perfusion in the cortex and white matter, which is atypical for an infant. The centrally located anechoic structures represent the paired lateral ventricles. (**C**) An image of the same 10-month-old 1 week later shows diffusely decreased brain perfusion, as well as enlarged paired lateral ventricles and the more medially located third ventricle. (**D**) Midcoronal image of a 6-month-old 3 h after cardiac arrest demonstrates minimal brain perfusion. Reproduced with permission from the journal Pediatrics, Vol. 143, Copyright © 2019 by the AAP.

The evaluation of HIE on CEUS is a continuing topic of interest made challenging by the variability in manifestations of HIE pathologically and radiologically. Gray- and white-matter perfusion can be differentially affected in HIE and stroke [18,19,20]. Additionally, there are multiple patterns of HIE, including peripheral or watershed and the more severe central or basal ganglia-thalamic [20] (Figure 3). Multiple quantitative approaches to assessing this injury have been proposed, including central gray-cortex perfusion ratio [14]. In normal neonates, the basal ganglia are perfused more than cortical gray matter or white matter [21]. In an early central pattern of HIE, the central gray nuclei demonstrate hyperperfusion, and the ratio of basal ganglia to white-matter perfusion would be elevated; this ratio would be decreased in early peripheral HIE with the cortex demonstrating relative hyperperfusion [14]. The differentiation between normal and abnormal ratios is challenging in mixed central and peripheral injury patterns, however, and the availability of normative brain CEUS data may be helpful in this regard. Moreover, while initial phases of HIE are characterized by early hyperperfusion, there is often a later transition to hypoperfusion [22]. Hyperperfusion may persist for a longer time or transition to hypoperfusion in some individuals as the injury from HIE progresses. The clinical significance of the variability in HIE evolution is not clear and requires more investigation. The stage of HIE could, therefore, affect both quantitative and qualitative evaluation of HIE. On the other hand, the dynamic evolution of injury can be assessed in a serial manner using CEUS. Additional work is needed to determine the short- and long-term prognostic value of CEUS in HIE and to determine optimal times of imaging. CEUS can also detect cessation of blood flow in the brain [5]. In the future, with further validation, CEUS may also play a role in providing supplemental evidence in suspected cases of brain death.

**Figure 4:**
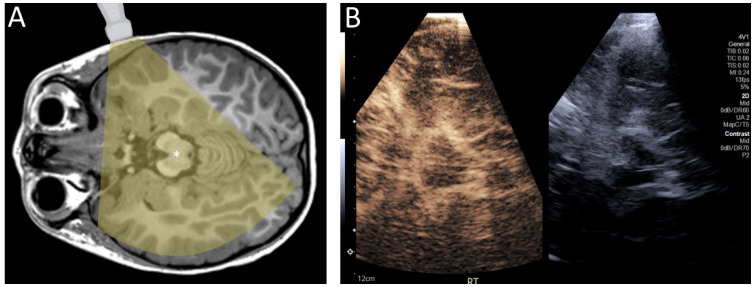
(**A**) Diagram demonstrating the transtemporal approach for ultrasound. (**B**) Contrast-enhanced ultrasound of the brain of a 4 year old child through a transtemporal window. In infants, ultrasound can be performed through open fontanelles. In older individuals, including children and adults, ultrasound can be performed through the transtemporal window.

While most of these studies have focused on CEUS in infants, which are an obvious target population for CEUS because of their fontanelles, CEUS may be used in older children and adults through a transtemporal window (Figure 4). The squamous part of the temporal bone is thin and often allows for the sufficient penetration of sound energy to permit ultrasound imaging. Although attenuation by bone can partially limit visualization of intracranial structures, the use of contrast allows visualization of intracranial microvessels as compared to transcranial Doppler, which is limited to macrovascular imaging [23,24]. This has several theoretical uses, including evaluation of stroke [25,26,27,28,29], more subtle perfusion changes in the setting of arterial stenosis [30], and brain death [24]. Other windows, such as transcondylar, suboccipital, and transforaminal through the foramen magnum may increase the probability of intracranial macro- and microvascular flow assessment in cases where the transtemporal approach is insufficient.

Real-time, dynamic evaluation of brain perfusion on CEUS may prove useful in situations where maintaining brain perfusion is critical. The use of brain CEUS to monitor brain perfusion during congenital heart surgery has been described [31]. Following a similar principle, CEUS may theoretically be used to monitor brain perfusion during cardiopulmonary resuscitation (CPR) or extracorporeal membrane oxygenation (ECMO). In CPR and ECMO, the primary goal is the maintenance of brain perfusion. CEUS could be used during CPR to direct resuscitation strategies, including to evaluate the adequacy of chest compressions, monitor collateral brain flow, or direct medication administration [32]. Since CPR requires continuous evaluation of brain perfusion during resuscitation, an infusion rather than bolus of contrast would be preferred. Unlike optical technologies such as near infrared spectroscopy, CEUS acquisition and interpretation are feasible in the setting of motion degradation such as in active chest compressions. In ECMO, brain perfusion evaluation by CEUS could similarly be used to adjust ECMO settings and guide medication use [33].

Other potential uses for CEUS in critical care include evaluation of changes in perfusion surrounding intracranial hemorrhage, in cases of hydrocephalus and post cardiac arrest. Areas of active parenchymal hemorrhage would be expected to hypoenhance compared to normal brain parenchyma, allowing delineation of blood products and surrounding injured tissue. Brain perfusion may serve as a surrogate measure of intracranial pressure in hydrocephalus [13]. The Monro–Kellie hypothesis describes the relationship among the volume brain tissue, blood, and cerebrospinal fluid within the fixed volume of the skull. In the setting of hydrocephalus, the increased cerebrospinal fluid displaces brain tissue and blood, which may result in perfusion changes that can be evaluated on CEUS. In a piglet model of cardiac arrest, intracranial pressure and wash-out slope and peak enhancement were correlated immediately post return of spontaneous circulation (ROSC) and 3 h after ROSC, respectively [34]. CEUS may also be useful for identifying parenchymal injuries and edema in traumatic brain injury. In addition, CEUS can detect venous occlusion, such as of the dural venous sinuses, where slow flow is difficult to measure on Doppler [35].

## 3. Microvascular Imaging

Conventional color and power Doppler ultrasound have long demonstrated their utility in detecting large-vessel, high-velocity flow. To reduce the detection of background tissue motion or clutter, conventional Doppler utilizes a unidimensional wall filter to remove low frequencies and improve signal-to-noise ratio. This reduces Doppler’s sensitivity to slow flow and flow in the microvasculature.

MVI ameliorates this flaw of conventional Doppler by using a multidimensional wall filter that preserves signal from slow flow while removing clutter [36]. It shares the portability benefit of contrast-enhanced ultrasound but is currently available on fewer commercial ultrasound devices than CEUS-compatible modes. On the other hand, MVI does not require the use of intravascular contrast, which, while widely regarded as safe in the pediatric population, is associated with rare contrast reactions and cannot be used in patients with hypersensitivity to its components. Monochrome MVI subtracts the static background data, revealing only the flow in vasculature, and color MVI, like existing Doppler methods, superimposes color-coded flow on a background grayscale image. The implementation and the name of MVI vary by ultrasound device manufacturer, and not all ultrasound machines and probes currently have MVI. As an advanced form of Doppler, MVI carries no unique contraindications. Like other ultrasound techniques, MVI can be performed through a fontanelle or through other windows, such as transtemporal. Both curved and linear transducers may be used for MVI evaluation, with the latter yielding higher spatial resolution of near-field structures.

There are limitations to MVI that must be taken into consideration by the operator. MVI is affected by the size of the region of interest. If a region of interest is too large, the sensitivity for flow is decreased, even with MVI’s enhanced sensitivity compared to color and power Doppler ultrasound. In addition, MVI, like other Doppler techniques, is susceptible to flash artefacts if the gain is set too high. Similar to Doppler, MVI is highly motion-sensitive and a minimum 2 s pause between image planes is desirable to reduce motion-related artefacts.

Interpretation of MVI can be both qualitative and quantitative. Like CEUS, MVI can demonstrate areas of relative hyper- and hypoperfusion. Methods of quantification for MVI, while not explored in depth in literature, can reflect those of color and power Doppler [37,38,39,40]. One proposed method of quantification involves the calculation of mean pixel grayscale intensity of a region of interest in a monochrome MVI image [41].

**Figure 5:**
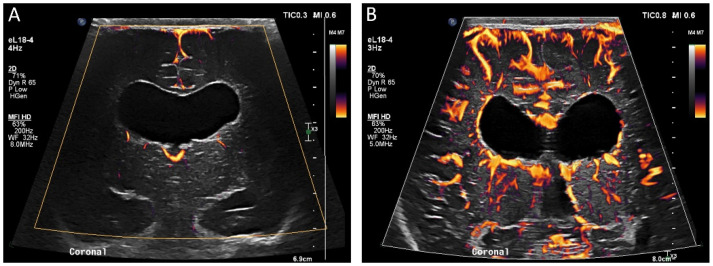
(**A**) Ultrasound with color microvascular imaging in a 6 day old, former 32 week 2 day infant with multiple congenital anomalies including absence of the septum pellucidum. Overall reduced cortical and deep gray nuclei microvascular flow is observed. (**B**) Ultrasound with color microvascular imaging of a 51 day old, former term infant with congenital hydrocephalus. Overall increased microvascular perfusion is seen throughout the cortex, white matter, and deep gray matter compared to the infant in (**A**). The significance of these and similar findings has yet to be elucidated and may be of import for clinical management and prognostication.

**Figure 6:**
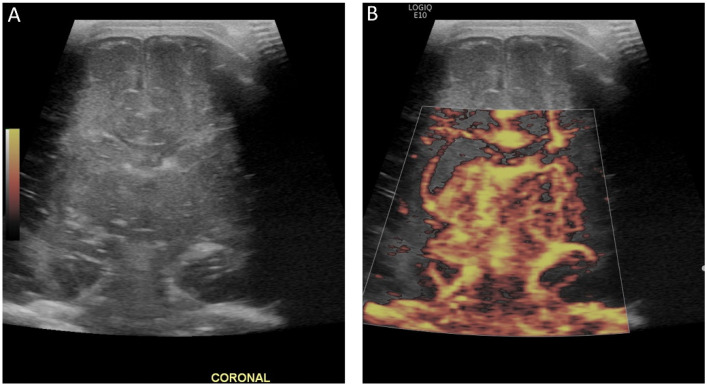
(**A**) Grayscale and (**B**) color microvascular imaging ultrasound a 13 day old, former 33 week 5 day infant with congenital heart disease and hypoxic respiratory failure on extracorporeal membrane oxygenation with seizures. Elevated flow is seen within the deep gray matter. This may reflect perfusion alterations in the setting of seizure, dysfunctional autoregulation, and/or evolving injury.

The use of MVI in the brain is in its early experimental stages. The few studies examining this proposed use of MVI demonstrate high inter-reader reliability for determining the presence of superficial and deep microvessels and depicting functional alterations in the brain with pathologic implications otherwise unavailable on grayscale ultrasound [42,43] (Figure 5 and Figure 6). Like on CEUS, changes in perfusion associated with ischemia may be visualized by MVI [43]. Furthermore, the detectability of superficial microvessels increases with gestational age in preterm infants, potentially allowing for evaluation of brain maturity [43]. In one study in older adults, MVI showed promise in the detection of acute ischemic stroke with reduced vascularity in areas of ischemia [44]. In instances of intraparenchymal intracranial hemorrhage, MVI may be used to delineate the margins of the hemorrhage, with acute blood products being avascular or hypovascular relative to normal brain tissue [45]. In addition to identifying the absence of microvasculature, MVI can demonstrate abnormal or increased microvascular perfusion, such as in the detection of abnormal vessels in brain tumors [46].

**Figure 7:**
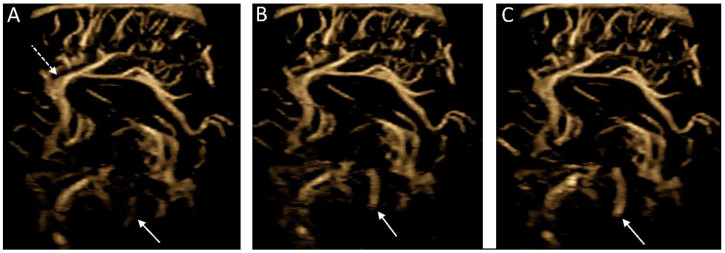
Microvascular imaging of the brain acquired in a in a midsagittal plane. (**A**–**C**) Consecutive images were acquired demonstrating cerebrospinal fluid flow within the cerebral aqueduct (solid arrows). Note is made of the anterior cerebral artery and its branches (dashed arrow).

Another novel use of MVI is the detection of cerebrospinal fluid flow, the velocity of which is often below the threshold of typical Doppler imaging (Figure 7). One case study has successfully used MVI to demonstrate cerebrospinal fluid flow in post-hemorrhagic hydrocephalus [47]. Further work is needed to understand the nature of flow (i.e., velocity, turbulence, composition) detectable using MVI such that nonvascular applications of MVI be applied for diagnostic and/or prognostic utility.

## 4. Elastography

Sonographic elastography is an advanced ultrasound technique that evaluates tissue stiffness. A value called the elastic or Young’s modulus reflects the elasticity of a tissue, with easily deformed tissues having a lower modulus. The two primary approaches to sonographic elastography are strain or compressive and shear wave elastography (SWE). In strain or compression elastography, manual compression by the ultrasound user or internal physiologic motion deforms the tissue, and the resultant displacement of tissue is used to calculate Young’s modulus. A lower displacement denotes greater tissue stiffness and a greater Young’s modulus. In SWE, a high-intensity pulse or acoustic radiation force is transmitted into tissue to generate shear waves perpendicular to the axis of the initial force. A greater shear wave speed denotes greater tissue stiffness and a greater Young’s modulus.

While sonographic elastography is better known for its use in breast, liver, prostate, thyroid, and other tissues and is approved in the United States for evaluation of abdominal organs, SWE has recently been used off-label to evaluate tissue stiffness of the brain parenchyma [48,49,50]. Early studies have shown that SWE is technically feasible in infants, and that different regions of the infant brain possess different levels of elasticity [50,51,52]. Most studies have focused on evaluating the stiffness of periventricular white matter, cortex, and the deep gray nuclei. These results are also reproducible [52]. Elastography can also be performed through a transtemporal window [53,54].

Potential adverse effects of sonographic elastography in the brain are unknown, including in the pediatric population, and additional research is needed to confirm its safety profile. Elastography uses a higher thermal index than conventional ultrasound, although still within guidelines for ultrasound safety [55]. However, bone has a greater rate of heat deposition than brain, and adjacent brain is, therefore, at risk of indirect heating [56]. In a neonatal mouse model, there were short-lived alterations in neuronal gene expression at 24 h after exposure to SWE, but these changes did not persist at 3 months [57]. No human studies have reported the adverse effects of SWE, noting that the literature on SWE is limited at this time. Additional preclinical and clinical studies are needed to evaluate its safety.

Physiologic processes can increase or decrease tissue stiffness, and this is also true in the brain. Gray-matter stiffness is lower in preterm than term neonates [52]. This, like visibility of superficial microvessels, may offer information regarding brain maturity in preterm infants. Differences in stiffness between preterm and term neonates may be related to myelination, neuronal growth, increased synapse formation, or propagation of glial cells [58]. Normative data are not yet available for stiffness or elasticity of different regions of the pediatric brain. It is important to note that anisotropy can affect these results; therefore, technique and orientation of imaging must be standardized in order to produce normative data. In SWE of muscle fibers, the relative orientation of the ultrasound probe and muscle fibers affects the measured elasticity [59].

Pathology also affects the stiffness of brain parenchyma. One study in neonates found an association between intraparenchymal hemorrhage and increased adjacent white and deep gray nuclei stiffness [52]. A study comparing ischemic and hemorrhagic stroke found increased stiffness within the region of infarct [60]. A separate rodent study investigating the effect of traumatic brain injury and hemorrhage on stiffness found the opposite, with brain injury and hemorrhage correlating with decreased stiffness at 24 h post injury [61]. The former study did not disclose the timeline between hemorrhage and elastography, and the disparity between the results may be related to the timing of injury. Elastography of the hemisphere contralateral to the intracranial pathology may provide information on degree of mass effect and midline shift [60]. Other changes, such as periventricular leukomalacia, may alter tissue stiffness in a manner detectable by elastography.

One of the largest bodies of literature regarding the use of sonographic elastography of the infant brain is in the context of stroke. In mice models, brain tissue stiffness decreases in the affected hemisphere in the hours and days after middle cerebral artery occlusion, likely because of liquefactive necrosis and edema [62,63]. A reduction in blood flow may be responsible for modulus increases in the contralateral brain [63]. In contrast, a rat model of hypoxic–ischemic injury identified elevated tissue stiffness after ischemic insult [64]. The differences between these studies may be related to differences in technique; Martin et al. [62] and Xu et al. [63] occluded the middle cerebral artery for 2 h and 45 min, respectively, while Wang et al. [64] exposed rats to common carotid ligation for 72 h.

**Figure 8:**
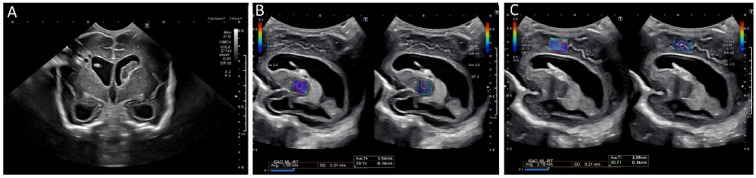
Ultrasound elastography of a 2 month old, former 28 week and 4 day infant with posthemorrhagic hydrocephalus after shunt placement. (**A**) A grayscale image demonstrating a right shunt catheter terminating in the right frontal horn. Periventricular cystic changes are seen related to prior infarct. Elastography measurements were taken over (**B**) the right basal ganglia, with values of 1.55 m/s and (**C**) the periventricular white matter with values of 2.18 m/s (**C**). These images were initially published in Pediatric Neurology, Volume 86, by authors Danielle deCampo MD PhD and Misun Hwang MD in the article “Characterizing the neonatal brain with ultrasound elastography,” pages 19–26, Copyright Elsevier (2018).

Brain tissue stiffness is also affected by hydrocephalus (Figure 8). In a prospective study using SWE, the brain stiffness of healthy neonates was compared to that of neonates with hydrocephalus [65]. Hydrocephalus was associated with greater tissue stiffness, and SWE measurements were positively correlated with intracranial pressure [65]. Transtemporal SWE in adults shows a similar correlation between stiffness and intracranial pressure [54], and this may be a viable approach in children with closed fontanelles.

The clinical significance of brain stiffness changes in various neurologic diseases has yet to be elucidated. The elasticity and stiffness of tissue may provide prognostic information regarding the evolution of hypoxic–ischemic injury or intracranial hemorrhage. While hydrocephalus, mass effect, and midline shift can be anatomically visualized with cross-sectional imaging, elastography may allow for evaluation of the physiologic effects of these processes and lead to changes in clinical management.

Ultrasound elastography may assist neurosurgeons with intraoperative detection of brain neoplasms and epileptogenic foci. SWE is more sensitive than surgeons at detecting residual neoplasm during resection [66,67]. In addition, SWE may allow for differentiation of gliomas of different grades and metastases. Low-grade gliomas and metastases were found to be stiffer than high-grade gliomas and normal brain parenchyma [55]. In one case report, a type II focal cortical dysplasia was localized during surgery using SWE on the basis of increased stiffness at the site of the lesion [68]. The lesion was not visualized on 3 T MRI, but identified on electroencephalography, positron emission tomography, and magnetoencephalography [68]. In the future, a combinatory approach applying CEUS and elastography may be adopted to better characterize brain lesions and guide interventions in real time.

## 5. Conclusions

Ultrasound is a pillar of pediatric imaging, providing visualization of pathology at the bedside, affordably and safely. This is particularly important in pediatric critical care, where logistical challenges often limit access to cross-sectional imaging. While highly useful, conventional ultrasound, both grayscale and Doppler, is limited in the breadth of information it can provide. Grayscale images offer only an anatomic perspective without functional data. Doppler, while functional, is not sensitive to slow flow.

Advanced ultrasound techniques, including CEUS, MVI, and sonographic elastography, provide functional information that can expand our understanding of physiologic and pathologic processes and complement existing techniques such as grayscale ultrasound and transcranial Doppler. With further research, these advanced ultrasound techniques may help to guide therapy and facilitate prognostication. Both CEUS and MVI provide unprecedented visualization of the microvasculature in the brain, allowing for bedside evaluation of processes affecting brain perfusion, such as stroke and hypoxic–ischemic encephalopathy. Elastography offers supplementary information regarding changes in brain tissue during these processes. This information was previously only obtainable through MRI. Creative uses of these techniques, such as the detection of cerebrospinal fluid flow by MVI, may take the place of similar MRI techniques in the critical care unit.

While these techniques are technically feasible and scientifically intriguing, most information available regarding their use in the brain is limited to animal models and case series. A lack of large prospective preclinical and clinical trials currently inhibits their widespread adoption. Additional studies are needed to assess the real-world clinical value of these techniques and spur widespread adoption as appropriate.
